# Unlocking new possibility of Fe_3_O_4_@C@Ag nanostructures as an advanced SERS substrate for ultrasensitive detection of low-cross-section urea biomolecules†

**DOI:** 10.1039/d4ra07487d

**Published:** 2025-01-06

**Authors:** Quan-Doan Mai, Dang Thi Hanh Trang, Dong Thi Linh, Nguyen Trung Thanh, Bui Hanh Nhung, Ong Van Hoang, Ta Ngoc Bach, Nguyen Quang Hoa, Anh-Tuan Pham, Anh-Tuan Le

**Affiliations:** a Phenikaa University Nano Institute (PHENA), Phenikaa University Hanoi 12116 Vietnam doan.maiquan@phenikaa-uni.edu.vn; b Faculty of Materials Science and Engineering, Phenikaa University Hanoi 12116 Vietnam tuan.leanh@phenikaa-uni.edu.vn; c University of Transport Technology Trieu Khuc, Thanh Xuan District Hanoi Vietnam; d Institute of Materials Science (IMS), Vietnam Academy of Science and Technology 18 Hoang Quoc Viet Hanoi 10000 Vietnam; e Faculty of Physics, VNU University of Science, Vietnam National University Hanoi, Thanh Xuan Hanoi Vietnam; f Faculty of Biotechnology, Chemistry and Environmental Engineering Hanoi 12116 Vietnam

## Abstract

Surface-enhanced Raman spectroscopy (SERS) is widely recognized as a powerful analytical technique, offering molecular identification by amplifying characteristic vibrational signals, even at the single-molecule level. While SERS has been successfully applied for a wide range of targets including pesticides, dyes, bacteria, and pharmaceuticals, it has struggled with the detection of molecules with inherently low Raman scattering cross-sections. Urea, a key nitrogen-containing biomolecule and the diamide of carbonic acid, is a prime example of such a challenging target. Found in human urine and blood, urea serves as an essential biomarker for diagnosing kidney dysfunction, liver disease, and heart failure, while its residue in water poses significant health risks. However, due to its low Raman cross-section, SERS has faced challenges in achieving high sensitivity for urea detection, limiting its potential in diagnosis and residual analysis. In this study, we present Fe_3_O_4_@C@Ag nanostructures as an advanced SERS substrate engineered for ultrasensitive urea detection. Our results reveal that Fe_3_O_4_@C@Ag nanostructures enable the detection of urea with a good limit of 5.68 × 10^−9^ M and a high enhancement factor of 3.67 × 10^6^. In addition, the substrate demonstrated high reliability, with repeatability and reproducibility showing relative standard deviations below 10%. Furthermore, the practicality of the Fe_3_O_4_@C@Ag nanostructures was evaluated in real-world scenarios using artificial urine and tap water samples as representative matrices for early disease diagnosis and water quality monitoring. The sensor successfully detected urea across concentrations as low as 10^−8^ M, with excellent recovery rates ranging from 90% to 99%, even in complex sample environments. These results highlight the remarkable sensitivity and versatility of Fe_3_O_4_@C@Ag nanostructures, overcoming SERS's traditional limitations in urea detection and unlocking new possibilities for clinical diagnostics and environmental monitoring.

## Abstract 2

In this study, we developed an advanced Fe_3_O_4_@C@Ag nanostructure-based SERS substrate, enabling ultra-sensitive, reliable, and practical detection of the low-Raman-cross-section biomolecule, urea.

## Graphical abstract

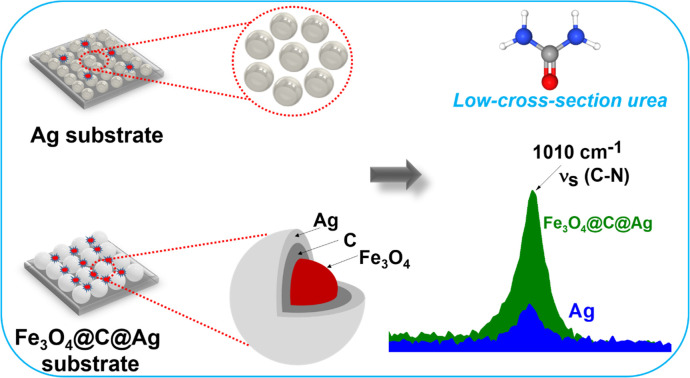

## 1. Introduction

Surface-enhanced Raman spectroscopy (SERS) is a pivotal technique in chemical and biological analysis, offering the ability to provide molecular fingerprint information with ultrasensitive detection (10^−13^ M to 10^−15^ M), even at the single-molecule level.^1–4^ Owing to its ultra-high sensitivity, label-free analysis, and rapid detection capabilities, SERS has found applications in critical fields such as food safety,^5^ environmental monitoring,^6^ surface and interface chemistry,^7^ biomedicine,^8^ and early disease diagnosis.^9^ At the heart of SERS are nanostructured materials based on noble metals such as Ag, Au, and Cu, where the Raman signals of target molecules are enormously amplified through two widely accepted mechanisms: electromagnetic and chemical.^1,2,10^ In recent decades, a vast amount of research has been dedicated to designing and developing nanomaterials for SERS, yielding significant breakthroughs. Nanomaterials ranging from simple colloidal^11,12^ and self-assembled nanoparticles^13,14^ to more sophisticated structures like composites^15,16^ and core–shell nanostructures^17,18^ have been engineered to maximize sensitivity across various analytes. These materials have demonstrated high SERS sensing efficiency in diverse applications, including detecting pesticides in food safety,^5^ organic dyes in environmental monitoring,^6^ and bacteria in biomedical studies.^8^ Despite these advances, a major challenge remains: the detection of molecules with inherently low Raman cross-sections.^19^ The Raman cross-section quantifies the probability of a molecule scattering light inelastically, with its magnitude reflecting the strength of the Raman signal.^20^ This is expressed in cm^2^ per sr per molecule. Molecules with high Raman cross-sections, such as rhodamine 6G, produce strong Raman signals, making them easier to detect, with typical values around 10^−25^ cm^2^ per sr.^21,22^ In contrast, molecules with low Raman cross-sections, such as small molecules (*e.g.*, gases and urea, with typical values around 10^−30^ cm^2^ per sr), exhibit weak Raman scattering, presenting significant challenges for detection.^23,24^ The nature of these molecules results in minimal Raman scattering enhancement under SERS conditions, which severely limits detection sensitivity, typically around 10^−3^ M to 10^−4^ M.^19,25^ Therefore, developing strategies to achieve high sensitivity for low Raman cross-section molecules is highly desirable, as it could significantly broaden the scope of SERS applications in critical fields.

Urea, the diamide of carbonic acid, is a key nitrogen-containing compound and an essential biomarker found in human urine and blood, with typical concentrations ranging from 2.5 × 10^−3^ M to 7.5 × 10^−3^ M in blood and 170 × 10^−3^ M to 590 × 10^−3^ M in urine, playing a crucial role in diagnosing kidney, liver, and heart diseases.^26,27^ Elevated blood urea levels exceeding 7.5 × 10^−3^ M (hyperuremia) may indicate kidney dysfunction, while levels below 2.0 × 10^−3^ M can be associated with severe liver damage or malnutrition. In urine, abnormally low urea concentrations below 100 × 10^−3^ M may suggest impaired renal function, whereas levels exceeding 600 × 10^−3^ M could indicate excessive protein metabolism or metabolic disorders. Beyond its significance in diagnostics, the analysis of urea residues in water or food also holds important health implications.^28,29^ Urea contamination in water and food often results from agricultural practices involving fertilizers or the illicit use of urea as an additive in food processing, posing risks to human health even at trace concentrations. Such urea residue concentration can increase the burden on the kidneys, potentially leading to kidney failure, or transform into nitrosamines in the body, particularly when combined with other pollutants, thereby elevating the risk of gastrointestinal cancers. Current techniques for detecting urea include enzymatic assays, colorimetric methods, and chromatographic techniques such as high-performance liquid chromatography (HPLC).^29,30^ These methods, while effective in specific scenarios, often suffer from limitations such as low sensitivity, interference from complex sample matrices, labor-intensive procedures, and the need for sophisticated instrumentation. In contrast, SERS offers ultra-high sensitivity, down to the single-molecule level, label-free detection, and the ability to detect urea in complex biological and environmental matrices with minimal preparation, making them particularly valuable for applications in clinical diagnostics, food safety and water quality monitoring. These distinctive benefits position SERS as a transformative approach compared to traditional methods. Unfortunately, the detection of urea using SERS remains challenging due to its inherently low Raman cross-section, resulting in weak signal enhancement. However, the ultra-high sensitivity and versatility of SERS drive efforts to address this limitation. Recent advancements have focused on enhancing the Raman signal through designed nanomaterial, enabling more effective detection of urea even in complex biological and environmental matrices. These developments hold significant promise for leveraging SERS as a practical tool in diverse applications, bridging the gap left by conventional methods. Chen *et al.* designed highly ordered Au/Cu hybrid nanostructure arrays with a high density of hotspots – areas between noble metal nanoparticles where strong electromagnetic fields are generated. This dense hotspot distribution enabled the SERS substrate to detect urea at concentrations as low as 1.0 × 10^−3^ M with excellent reproducibility.^25^ However, despite the high hotspot density, the detection sensitivity remains limited. Li and colleagues took a different approach by increasing the porosity of the SERS substrate to enhance the adsorption of urea molecules, bringing them closer to the nanostructured surface, which in turn improved the SERS signal. They fabricated a novel SERS substrate composed of silver and porous gold nanoparticles, providing additional enhancement from the gold's porous structure.^31^ This substrate achieved a detection limit of 1.0 × 10^−3^ M under biophysical conditions. In 2022, we introduced a photo-induced enhanced Raman scattering (PIERS) technique applied to Ag/TiO_2_ nanocomposite SERS substrates to improve urea detection by pre-irradiating the substrate with UV light.^32^ This pre-irradiation step significantly improved the performance of the Ag/TiO_2_ nanocomposite, enabling urea detection down to 4.6 × 10^−6^ M. This technique also enables the detection of urea residues in food (milk samples) at concentrations down to 10^−5^ M. Nevertheless, for an ultrasensitive sensing platform like SERS, there is still significant potential to further enhance and optimize the detection sensitivity for urea.

To achieve strong SERS enhancement for urea detection, it is essential that the design of the SERS substrate ensures a high density of hotspots. More importantly, urea molecules need to be effectively drawn to these hotspot regions to maximize the enhancement of Raman scattering – a feature we describe as “accessible hotspots”. However, this is challenging due to spatial constraints at the hotspots, where analyte molecules tend to accumulate on the outer surface of the material instead of at the hotspot sites. Thus, designing a SERS substrate that simultaneously provides both “dense hotspot density” and “accessible hotspots” is crucial for enhancing the sensitivity towards urea. In this study, we successfully designed and fabricated Fe_3_O_4_@C@Ag nanostructures as an advanced SERS substrate, combining both “dense hotspot density” and “accessible hotspots”. By reducing AgNO_3_ directly onto the surface of Fe_3_O_4_@C, Ag nanoparticles (NPs) were uniformly deposited, forming evenly distributed interparticle gaps around the Fe_3_O_4_@C spheres. This generated a dense three-dimensional network of hotspots within the Fe_3_O_4_@C@Ag structure. Furthermore, the adsorption ability of the carbon layer allows urea molecules to be efficiently adsorbed. Notably, the regions where the carbon layer adsorbs urea are precisely at the interparticle gaps formed by the Ag NPs – these are the hotspots, enabling the “accessible hotspots” feature of the Fe_3_O_4_@C@Ag nanostructures. As a result of these combined features, the Fe_3_O_4_@C@Ag nanostructures exhibit ultra-sensitive detection of urea, achieving a low detection limit of 5.68 × 10^−9^ M and an enhancement factor of 3.67 × 10^6^. The reliability of the Fe_3_O_4_@C@Ag nanostructures was demonstrated through repeatability and reproducibility tests, both yielding relative standard deviation (RSD) values below 10%, indicating high reliability. Additionally, the Fe_3_O_4_@C@Ag nanostructures demonstrated their practical applicability in real-world scenarios, with successful urea detection in artificial urine and tap water samples. As a proof of concept, the Fe_3_O_4_@C@Ag nanostructures effectively address the limitations of SERS in identifying and quantifying the urea, opening new avenues for research in disease diagnostics and environmental monitoring related to this critical biomarker.

## 2. Materials and methods

### 2.1. Materials

Ferrocene (Fe(C_5_H_5_)_2_, 98 wt%), hydrogen peroxide solution (H_2_O_2_, 30 v/v%), urea (CH_4_N_2_O, 99 wt%) and creatinine (C_4_H_7_N_3_O, ≥98%) were sourced from Sigma Aldrich (United Kingdom). Silver nitrate (AgNO_3_, ≥99.0 wt%), sodiumborohydride (NaBH_4_, 99 wt%), polyhexamethylene biguanide (PHMB, 99 wt%), potassium hydroxide (KOH, 96 wt%), ammonium nitrate ((NH_4_)_2_SO_4_, ≥99 wt%), sodium citrate (Na_3_C_6_H_5_O_7_, 99 wt%), ethanol (C_2_H_5_OH, 98 v/v%) and acetone (C_3_H_6_O, 99 v/v%) were purchased from Shanghai Chemical Reagent (China). All chemicals were used directly without further purification. Double distilled water was used throughout the experiments.

### 2.2. Synthesis of Fe_3_O_4_@C@Ag nanostructure materials and their characterizations

**Scheme 1:**
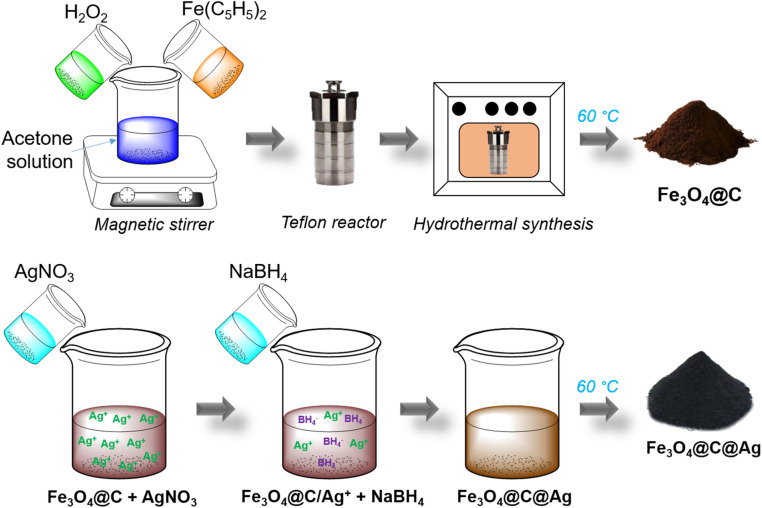
Schematic illustration of the synthesis process of Fe_3_O_4_@C and Fe_3_O_4_@C@Ag nanostructures.

The Fe_3_O_4_@C material was synthesized *via* a hydrothermal method using Fe(C_5_H_5_)_2_ as the precursor and H_2_O_2_ as the reducing agent. First, 0.8 g of Fe(C_5_H_5_)_2_ were evenly dispersed in 30 mL of C_3_H_6_O by magnetic stirring for 15 minutes. Next, 5 mL of H_2_O_2_ was gradually added to the solution, which was continuously stirred for an additional 30 minutes. This mixture was then transferred to a Teflon container, and the hydrothermal process was conducted for 20 h at 200 °C. The resulting product was washed three times with acetone using a high-speed centrifuge. Finally, the product was dried at 60 °C for 2 h to obtain Fe_3_O_4_@C powder, which was prepared for subsequent steps (Scheme 1). The Ag layer in the Fe_3_O_4_@C@Ag structure was formed by the reduction of AgNO_3_ using NaBH_4_. First, 50 mg of the Fe_3_O_4_@C material was evenly dispersed in 100 mL of double-distilled water by ultrasonic shaking for 5 minutes. Subsequently, 50 mg of AgNO_3_ was added to this mixture and stirred for 1 h to ensure the Ag^+^ ions were adsorbed onto the surface of Fe_3_O_4_@C. The Fe_3_O_4_@C/Ag^+^ material (with Ag^+^ ions adsorbed onto the surface of Fe_3_O_4_@C) was then recovered, and any excess Ag^+^ ions were removed to prevent the formation of Ag materials outside the Fe_3_O_4_@C surface. 23 mg of NaBH_4_ dispersed in 10 mL of double-distilled water was gradually added to the Fe_3_O_4_@C/Ag^+^ mixture to directly reduce the Ag^+^ ions on the surface of Fe_3_O_4_@C, leading to the formation of the Fe_3_O_4_@C@Ag material. The product was washed three times with acetone using a high-speed centrifuge. Finally, the sample was dried at 60 °C for 2 h to obtain powdered Fe_3_O_4_@C@Ag nanostructures, ready for further experiments (Scheme 1). The SERS substrate based on bare Ag NPs was prepared using Ag materials synthesized by the reduction of AgNO_3_ with NaBH_4_. First, 50 mg of AgNO_3_ was dispersed in 100 mL of double-distilled water by uniform magnetic stirring for 30 minutes. Next, 5 mg of the surfactant polyhexamethylene biguanide (PHMB) was added to the mixture to stabilize the Ag nanoparticles during their formation. Finally, 23 mg of NaBH_4_, dispersed in 10 mL of double-distilled water, was gradually added to the mixture to reduce Ag^+^ ions into Ag NPs. The reaction proceeded for 3 h, resulting in Ag NPs in the form of a colloidal solution.

The morphology of the Fe_3_O_4_@C, Fe_3_O_4_@C@Ag nanostructures and Ag NPs was examined using field emission scanning electron microscopy (FE-SEM, Hitachi S-4800) operating at an acceleration voltage of 5 kV. The elemental composition and distribution of the Fe_3_O_4_@C and Fe_3_O_4_@C@Ag materials were characterized using Energy-Dispersive X-ray (EDX) spectroscopy along with EDX mapping analysis. The crystalline properties of the Fe_3_O_4_@C and Fe_3_O_4_@C@Ag nanostructures were investigated using X-ray diffraction (Bruker D5005 X-ray diffractometer, Cu Kα, *λ* = 1.5406 Å) under a voltage of 40 kV and a current of 30 mA. The composition and chemical properties of the Fe_3_O_4_@C and Fe_3_O_4_@C@Ag nanostructures were analyzed using Raman spectroscopy (Horiba Macro-RAM™) with 785 nm laser excitation. The optical properties of Ag NPs, Fe_3_O_4_@C, and Fe_3_O_4_@C@Ag nanostructures were investigated using a JENWAY 6850 double-beam ultraviolet-visible (UV-Vis) spectrophotometer with 10 mm path length quartz cuvettes.

The UV-Vis spectra were used to evaluate the adsorption capacity of urea molecules on the surfaces of Ag NPs, Fe_3_O_4_@C, and Fe_3_O_4_@C@Ag materials. Initially, the UV-Vis spectrum of urea was recorded in the range of 200–800 nm to identify its characteristic absorption peaks. These characteristic peaks were then monitored over time during the adsorption process. The UV-Vis spectra were collected at various time intervals during the interaction between urea and the materials under uniform stirring, at time points such as 0, 5, 10, 15, and 20 minutes, with 0.1 g of material in 50 mL of solution containing urea at a concentration of 10^−4^ M. The adsorption efficiency was assessed at the time when the adsorption equilibrium state was reached, evidenced by no further decrease in the characteristic absorption peak of urea in the UV-Vis spectrum. This efficiency was calculated using the formula: adsorption capacity (%) = (1 − *I*_*t*_/*I*_0_) × 100, where *I*_*t*_ represents the intensity of the characteristic absorption peak of urea at time *t* when equilibrium adsorption is achieved, and *I*_0_ is the initial intensity of the absorption peak of urea at a concentration of 10^−4^ M.

### 2.3. Substrate preparation, SERS measurements

Aluminum (Al) substrates, measuring 1 cm × 1 cm × 0.1 cm, were fabricated with a surface-active area having a diameter of 0.2 cm by creating a small hole of the same size on the Al substrate surface. This active surface area was designed to deposit materials onto it and to prevent uneven deposition of these materials within the designated area. The substrates were thoroughly cleaned with ethanol and allowed to air dry at room temperature. Next, a solution containing 1 mg mL^−1^ of bare Ag NPs and Fe_3_O_4_@C@Ag nanostructures was prepared deposited onto the active surface area of the Al substrates using a drop-casting method, which were then dried at room temperature to ensure natural evaporation and to prevent uneven material deposition. This process was repeated for all SERS substrates.

Standard solutions of urea at various concentrations (10^−3^ to 10^−9^ M) were prepared using double-distilled water as the solvent. For each measurement, 5 μL of each urea concentration was directly applied to the prepared SERS substrates and allowed to evaporate under laboratory conditions. The SERS spectra of each substrate were collected immediately afterward using a MacroRaman™ Raman spectrometer (Horiba) with 785 nm laser excitation. Raman measurements were conducted using a 100× objective with a numerical aperture of 0.90. The laser power was set to 45 mW at a 45° contact angle, resulting in a diffraction-limited laser spot diameter of 1.1 μm (1.22*λ*/NA) and a focal length of 115 nm. Each measurement had an exposure time of 20 s with three accumulations, and the final spectrum was obtained after baseline calibration, which involves subtracting the elevated background signal to ensure the spectrum clearly displays the signal while maintaining the integrity of the recorded data.

### 2.4. Practicability evaluation experiment

The real samples used in this study were artificial urine and tap water, which were employed to evaluate the practicability of the Fe_3_O_4_@C@Ag nanostructure-based SERS sensor. The preparation of artificial urine was carried out as follows: to 1 liter of distilled water, the following reagents were added: 1 g of potassium hydroxide, 1 g of ammonium nitrate, 1 g of ammonium sulfate, 1 g of sodium citrate, and 0.5 g of creatinine. This artificial urine was prepared according to the procedure outlined in a previous study, with slight modifications.^33^ Urea was then added to achieve concentrations ranging from 10^−5^ to 10^−8^ M. Similar urea concentrations were also prepared in tap water samples collected from a residential area. These urea-containing samples were subsequently used directly for SERS signal detection without any further processing.

## 3. Results and discussion

### 3.1. Characterizations of Fe_3_O_4_@C@Ag nanostructure materials

**Fig. 1:**
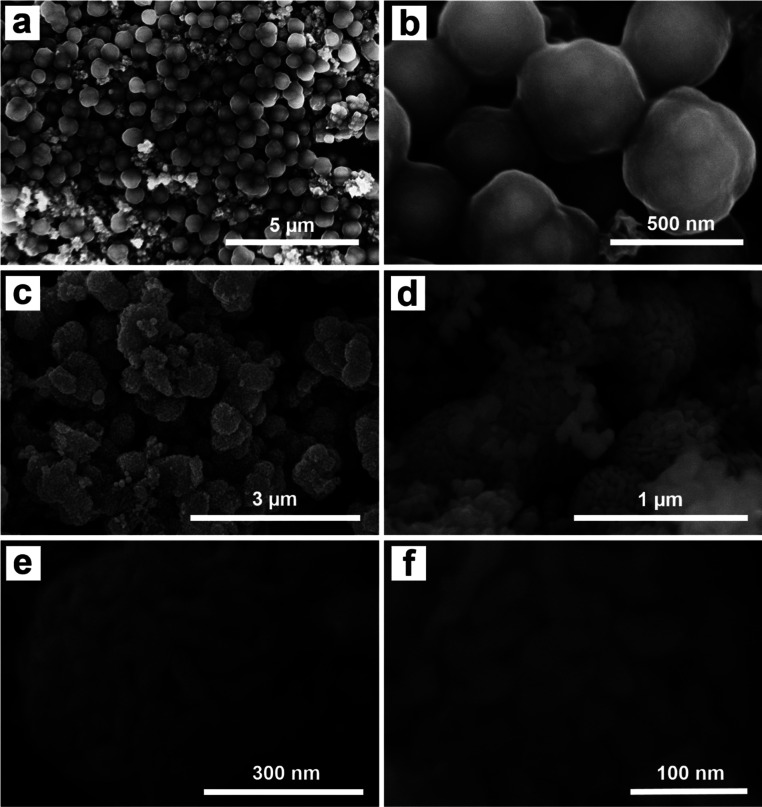
FE-SEM images of Fe_3_O_4_@C (a, b) and Fe_3_O_4_@C@Ag (c–f) at different magnification.

The morphology of the Fe_3_O_4_@C and Fe_3_O_4_@C@Ag materials was characterized using FE-SEM analysis, as illustrated in Fig. 1. Fig. 1a and b display FE-SEM images of Fe_3_O_4_@C at different resolutions. The successful synthesis of Fe_3_O_4_@C *via* a hydrothermal method utilizing ferrocene (Fe(C_5_H_5_)_2_) as a precursor has been established in previous studies.^34,35^ In this experiment, the hydrothermal process conducted at 200 °C for 20 hours resulted in spherical Fe_3_O_4_@C particles with an average diameter of 480 nm, demonstrating good uniformity (a detailed analysis of the distribution is provided in Fig. S1 of the ESI†). The surface is coated with a rough layer, likely composed of carbon, which facilitates the adsorption of Ag^+^ ions during synthesis. Furthermore, the Fe_3_O_4_@C particles show good dispersion without noticeable aggregation. This behavior is likely attributed to the magnetic properties of the Fe_3_O_4_ core, which assist in particle separation, stabilize the material, and effectively prevent aggregation. The separation of Fe_3_O_4_@C particles could facilitate a more uniform adsorption of Ag^+^ ions onto the surface of each particle, leading to the formation of a more uniform Ag layer in the Fe_3_O_4_@C@Ag structure. Fig. 1c and d show the FE-SEM images of the Fe_3_O_4_@C@Ag material. Notably, the overall size of the material appears to increase compared to Fe_3_O_4_@C with an average diameter of 610 nm (Fig. S2†). At lower magnification in Fig. 1c, the particle surfaces exhibit a significant transformation from a smooth to a porous texture. A closer examination in Fig. 1d reveals that a layer of nanoparticles envelops the Fe_3_O_4_@C surface. These nanoparticles are uniformly distributed and densely arranged in three dimensions around all the Fe_3_O_4_@C particles observed (such uniform formation on each particle may be due to the separation of the Fe_3_O_4_@C particles). This regular and compact arrangement creates gaps between the nanoparticles, which are extensively distributed throughout the sample. These particles are attributed to the Ag NP. Consequently, the Ag NP forms a protective layer around the Fe_3_O_4_@C, resulting in the Fe_3_O_4_@C@Ag nanostructure, characterized by a high density of interparticles (gaps between the Ag particles) that serve as “hotspots”. A closer examination of the single Fe_3_O_4_@C@Ag nanoparticle at higher resolution (Fig. 1e and f) provides clearer confirmation of the formation of numerous hotspot sites through the appearance of many interparticle positions between the Ag particles on the Fe_3_O_4_@C surface, where electromagnetic field interactions are strongest. Therefore, FE-SEM images confirm that the obtained Fe_3_O_4_@C@Ag nanostructure achieves the targeted feature of a “dense hotspot density”. For the bare Ag NPs materials, the FE-SEM images and size distribution histogram presented in Fig. S3† confirm that the synthesized Ag NPs have a spherical shape with relatively uniform sizes, and the average size was calculated to be 24 nm.

**Fig. 2:**
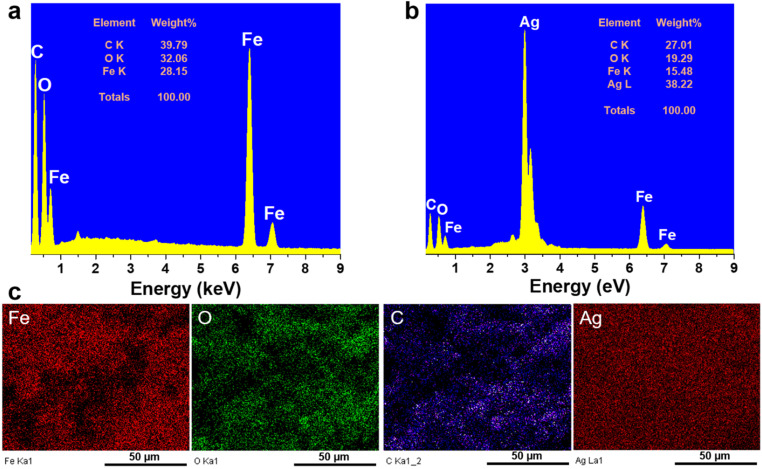
EDX spectra of Fe_3_O_4_@C (a) and Fe_3_O_4_@C@Ag (b); and EDX mapping analysis of Fe_3_O_4_@C@Ag (c).

Fig. 2 shows the results of EDX and EDX mapping measurements of the Fe_3_O_4_@C and Fe_3_O_4_@C@Ag samples to assess the presence and distribution of Fe_3_O_4_, C, and Ag components in the three-component Fe_3_O_4_@C@Ag nanostructure. Fig. 2a presents the EDX measurement results for the Fe_3_O_4_@C sample and confirms the presence of iron (Fe), oxygen (O), and carbon (C) elements, with respective contents of 28.15%, 32.06%, and 39.79%. Additionally, no foreign elements were observed in this sample. This result, combined with the FE-SEM image, indicates the formation of the Fe_3_O_4_@C material with high purity. The presence of Ag in the Fe_3_O_4_@C@Ag structure is confirmed through the EDX results shown in Fig. 2b. It can be observed that, in addition to the core Fe_3_O4@C elements of Fe, O, and C, there is also the presence of Ag with a content of 38.22%, and no foreign elements were observed, indicating the high purity of the Fe_3_O_4_@C@Ag sample formed. The EDX mapping analysis results in Fig. 2c confirm the distribution of the Fe_3_O_4_, C, and Ag components in the Fe_3_O_4_@C@Ag nanostructures. These four elements are evenly distributed within the observed area. Notably, the uniform distribution of the Ag element, observed in conjunction with the FE-SEM images, shows the formation and uniform distribution of the Ag layer within the Fe_3_O_4_@C@Ag structure.

**Fig. 3:**
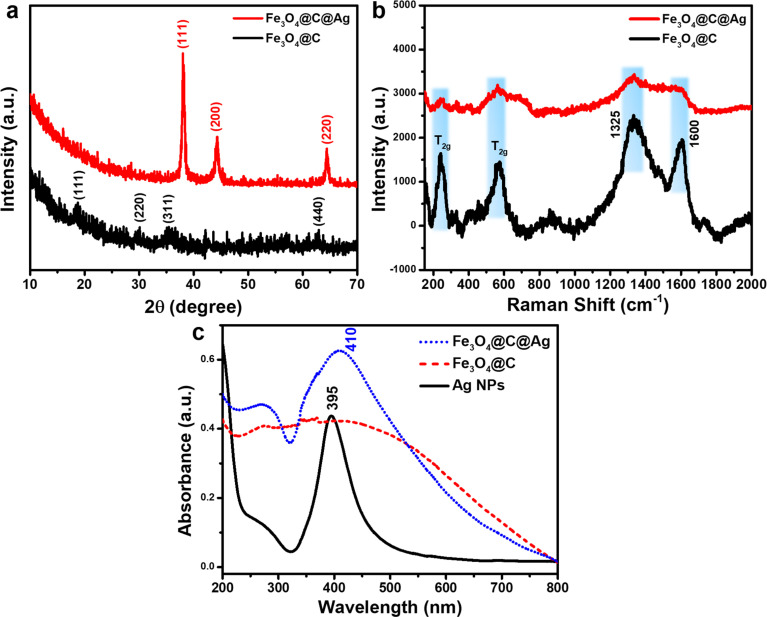
XRD patterns (a), Raman spectra (b) and UV-Vis spectra (c) of Fe_3_O_4_@C and Fe_3_O_4_@C@Ag.

The crystalline properties and composition of Fe_3_O_4_@C and Fe_3_O_4_@C@Ag materials were evaluated using X-ray diffraction (XRD) to verify the formation and structure of these designed materials. Fig. 3a presents the XRD patterns for Fe_3_O_4_@C and Fe_3_O_4_@C@Ag. The diffraction peaks corresponding to the crystallographic planes (111), (220), (311), and (440) are observed at 2 theta values of 18.80°, 29.93°, 35.31°, and 62.76°, respectively, aligning well with the characteristic diffractions of the Fe_3_O_4_ inverse spinel structure (JCDPS File No. 89-0691). However, the intensity of these peaks is weak and indistinct, likely due to the carbon layer surrounding the Fe_3_O_4_ particles, which interferes with the diffraction process. In the XRD pattern of the Fe_3_O_4_@C@Ag material, distinct peaks attributable to metallic Ag appear. Three prominent peaks at 2*θ* = 38.05°, 44.25°, and 64.50° correspond to the (111), (200), and (220) reflections of metallic Ag (JCDPS File No. 04-0783). The sharpness and high intensity of these peaks indicate a high degree of crystallinity in the formed Ag layer. Notably, the characteristic diffraction peaks of Fe_3_O_4_ are absent in the XRD spectrum of Fe_3_O_4_@C@Ag, suggesting that the presence of the carbon and Ag layers has completely suppressed the diffraction signal from the Fe_3_O_4_ core. Furthermore, no anomalous diffraction peaks are observed, confirming the high purity of the resulting material.

The Raman spectra were analyzed to complement the results obtained from FE-SEM imaging and XRD patterns, as illustrated in Fig. 3b. The Fe_3_O_4_@C material displays two prominent and broad peaks centered at approximately 1325 cm^−1^ and 1600 cm^−1^, corresponding to the D band and G band, respectively. The D band is associated with defects and disordered regions in the carbon structure (sp^3^ bonded), while the G band indicates in-plane stretching of ordered graphitic crystallites (sp^2^ bonded).^36^ The pronounced intensities of these peaks confirm the presence of a carbon layer in the Fe_3_O_4_@C structure. Furthermore, the higher intensity of the D band relative to the G band suggests a significant presence of defects and disorder, which increases the number of active sites available for Ag^+^ ion adsorption, facilitating the formation of Ag nanoparticles on the surface of Fe_3_O_4_@C. In addition, the carbon layer beneath the Ag nanoparticles provides additional active sites for ion storage, playing a crucial role in SERS.^37,38^ The gaps between the Ag NPs, as observed in the FE-SEM images, function as hotspots. The underlying carbon layer can attract urea molecules to these hotspots, effectively guiding them to optimal adsorption locations. This configuration results in “accessible hotspots” within the Fe_3_O_4_@C@Ag nanostructures. Moreover, two additional strong peaks at 243 cm^−1^ and 570 cm^−1^ are attributed to the T_2g_ vibrational mode of Fe_3_O_4_.^39,40^ The slight shift in the positions of these peaks compared to the characteristic scattering positions of uncoated Fe_3_O_4_ suggests a gravity interaction between the carbon shell and Fe_3_O_4_ core affecting in vibrational modes of the Fe_3_O_4_ material. Analysis of the Raman spectrum for Fe_3_O_4_@C@Ag reveals a reduction in the intensity of all peaks associated with both the Fe_3_O_4_ core and the carbon shell. This decrease may result from the shielding effect of the surrounding Ag NPs, which absorb or scatter the incident light, thereby reducing the signal from the Fe_3_O_4_@C core, as clearly seen in the FE-SEM images. Additionally, the absence of other scattering peaks indicates high sample purity. Notably, the weak appearance of scattering peaks from the substrate is advantageous for SERS applications, as it minimizes background noise and enhances the analysis during SERS measurements.

The UV-Vis spectra of Ag NPs, Fe_3_O_4_@C, and Fe_3_O_4_@C@Ag materials are shown in Fig. 3c to evaluate the optical properties of these samples. The absorption in the range of 350 nm to 450 nm, with a sharp peak at 395 nm for Ag NPs, is attributed to the resonant excitation of surface plasmons (SPR). In contrast, the Fe_3_O_4_@C material does not exhibit any characteristic absorption peaks but absorbs light over a broad range from 200 nm to 700 nm. The Fe_3_O_4_@C@Ag nanostructures display absorption behavior that combines the properties of both Fe_3_O_4_@C and Ag components. This material shows efficient absorption in the range from 350 nm to 500 nm, with a peak at 410 nm due to the SPR of the Ag layer. The peak shifts slightly to the red, broadens, and decays in intensity when compared to pure Ag NPs. This shift is attributed to the Fe_3_O_4_@C core, which increases the effective local dielectric constant of the Ag shell.^41^ Additionally, the Fe_3_O_4_@C@Ag nanostructures exhibit absorption in other regions from 200 nm to 700 nm due to the absorption of the Fe_3_O_4_@C core. The maintained SPR characteristics from the Ag NPs, combined with the enhanced local dielectric constant between the Ag and the Fe_3_O_4_@C core, promise strong SERS sensing capabilities for the Fe_3_O_4_@C@Ag nanostructures.

**Fig. 4:**
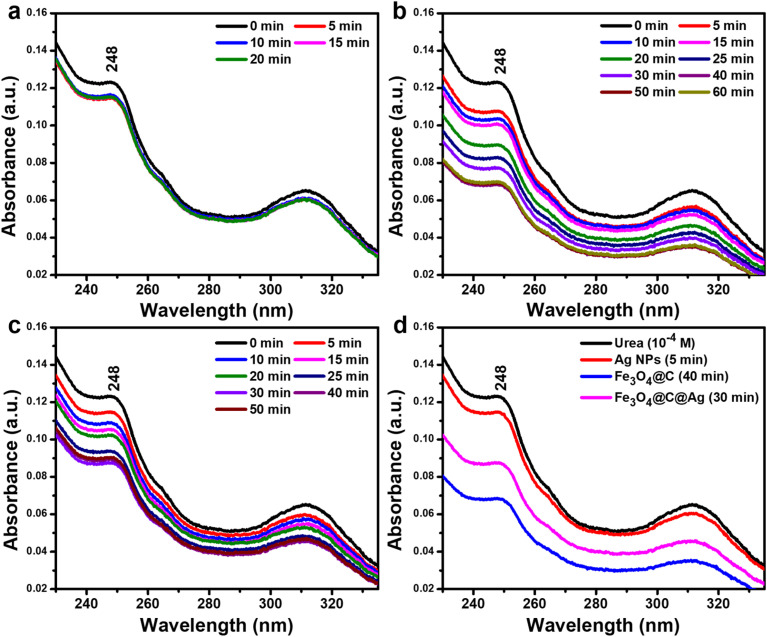
Evaluation of urea adsorption over time by monitoring the decrease in the intensity of the characteristic absorption peak at 248 nm for Ag NPs (a), Fe_3_O_4_@C (b), and Fe_3_O_4_@C@Ag (c). The comparison highlights the adsorption efficiency of urea across these materials at their respective equilibrium adsorption points (d).

The adsorption efficiency of Ag NPs, Fe_3_O_4_@C, and Fe_3_O_4_@C@Ag nanostructures for urea molecules was evaluated to clarify their adsorption behaviors and demonstrate the “accessible hotspots” feature of Fe_3_O_4_@C@Ag. Detailed experimental conditions are provided in the ESI.† UV-Vis spectra of urea (10^−4^ M), presented in Fig. S4,† exhibit characteristic absorption peaks at 248 nm and 312 nm. The adsorption process was monitored within the wavelength range of 230–330 nm by observing changes in the 248 nm peak intensity. Ag NPs showed limited urea adsorption, achieving only 8% efficiency after 5 minutes, with no further reduction in peak intensity over time, indicating rapid equilibrium (Fig. 4a). In contrast, Fe_3_O_4_@C demonstrated significantly higher adsorption capacity, with the peak at 248 nm decreasing steadily and reaching 44% efficiency after 40 minutes, where equilibrium was achieved (Fig. 4b). Fe_3_O_4_@C@Ag nanostructures showed intermediate performance, with 30% adsorption efficiency achieved within 30 minutes (Fig. 4c). The comparison in Fig. 4d reveals Fe_3_O_4_@C as the most efficient material for urea adsorption (44%), followed by Fe_3_O_4_@C@Ag (30%) and Ag NPs (5%). The superior adsorption efficiency of Fe_3_O_4_@C and Fe_3_O_4_@C@Ag is attributed to the high adsorption activity of the carbon layer. For Fe_3_O_4_@C@Ag, the Ag layer partially shields the carbon, slightly reducing adsorption efficiency. However, gaps in the Ag layer, observed *via* FE-SEM, create “accessible hotspots”, which effectively attract urea molecules. These accessible hotspots, combined with the dense hotspot density inherent to Fe_3_O_4_@C@Ag, enhance its potential for urea sensing. This dual feature underscores the material's suitability for high-performance urea detection.

### 3.2. SERS sensing performance of Fe_3_O_4_@C@Ag nanostructure substrate to detect urea

**Fig. 5:**
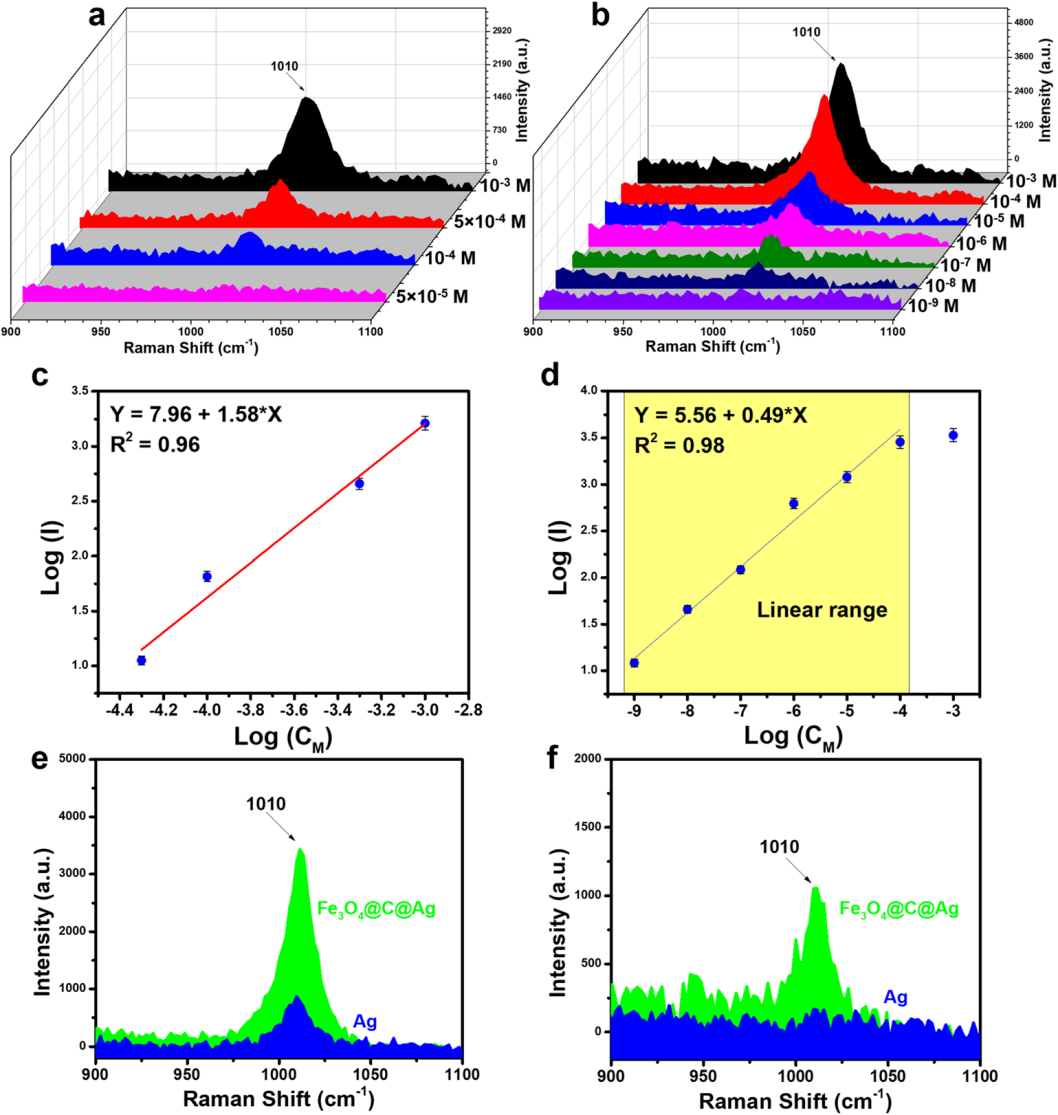
(a) SERS spectra of urea on bare Ag NPs in the concentration range of 10^−3^ to 5 × 10^−5^ M; (b) SERS spectra of urea on Fe_3_O_4_@C@Ag nanostructures in the concentration range of 10^−3^ to 10^−9^ M; (c) plot of the log of SERS intensity *versus* concentration at 1010 cm^−1^ on bare Ag NPs (slope 0.73 ± 0.02, intercept 5.46 ± 0.02); (d) plot of the log of SERS intensity *versus* concentration at 1010 cm^−1^ on Fe_3_O_4_@C@Ag (slope 0.59 ± 0.02, intercept 5.56 ± 0.02); comparison of SERS intensity of urea on Fe_3_O_4_@C@Ag and Ag NPs at concentrations of 10^−4^ M (e) and 10^−6^ M (f).

The SERS sensor efficiency for urea detection was evaluated using two types of SERS substrates: bare Ag NPs and Fe_3_O_4_@C@Ag nanostructures, aiming to compare their sensing performance. Urea, a simple organic molecule containing one carbonyl group (C=O) and two amino groups (–NH_2_), exhibits a Raman spectrum in its powdered state with a prominent scattering peak at 1020 cm^−1^, attributed to the C–N stretching mode vibrations in its molecular structure (Fig. S5†).^42^ The Raman spectrum of urea solution at a concentration of 10^−3^ M on a bare Ag substrate (without SERS materials) does not exhibit any characteristic peaks (Fig. S5†). Fig. 5a illustrates the SERS spectra of urea across a concentration range of 10^−3^ to 5 × 10^−5^ M on the bare Ag NPs. The SERS spectra of urea exhibits a single characteristic band at 1010 cm^−1^, slightly shifted from the Raman spectrum of the powder, and corresponding to the C–N stretching mode.^25,43^ This characteristic peak was clearly observed at a concentration of 10^−3^ M, with decreasing intensity at concentrations of 5 × 10^−4^ M and 10^−4^ M, ultimately disappearing entirely at 5 × 10^−5^ M. A linear relationship between the intensity of the peak at 1010 cm^−1^ and the corresponding concentrations was established through logarithmic transformation, as shown in Fig. 5c. The linear range obtained was from 10^−3^ M to 10^−6^ M, with the linear equation represented as *y* = 7.96 + 1.58 × *x*, yielding a linear correlation coefficient (*R*^2^) of 0.96. Based on the derived linear equation, the limit of detection (LOD) was calculated to be 7.68 × 10^−5^ M (details of the LOD calculation are provided in the ESI†).

The combination of two features – “dense hotspots density” and “accessible hotspots” – in the Fe_3_O_4_@C@Ag nanostructures substrate promises to enhance the sensor performance of this SERS substrate. Fig. 5b illustrates the SERS detection efficiency for urea using the Fe_3_O_4_@C@Ag nanostructures across a concentration range of 10^−3^ M to 10^−9^ M. The superior detection capability of the Fe_3_O_4_@C@Ag material is evident. Specifically, at concentrations of 10^−3^, 10^−4^, and 10^−5^ M, the peak at 1010 cm^−1^ is distinctly visible with high intensity and sharpness. In contrast, for the bare Ag NPs substrate, the signal for this peak completely disappears at a concentration of 5 × 10^−5^ M. The peak at 1010 cm^−1^ remains detectable even at a urea concentration of 10^−8^ M and only vanishes at an extremely low concentration of 10^−9^ M (see Fig. S6† to better observe the intensity decrease at these two concentrations, compared with the Raman spectrum of the bare Fe_3_O_4_@C@Ag substrate). A linear relationship between the logarithmic function of intensity and concentration was established, resulting in a wide linear range from 10^−4^ M to 10^−9^ M, described by the equation *y* = 5.56 + 0.49 × *x*, with a high *R*^2^ value of 0.98 (Fig. 5d). Based on this equation, the LOD was determined to be as low as 5.68 × 10^−9^ M. This detection limit for a molecule with a low Raman cross-section like urea, coupled with the extensive linear range from 10^−4^ M to 10^−9^ M and a high *R*^2^ value, underscores the excellent sensing capabilities of the Fe_3_O_4_@C@Ag SERS substrate. The SERS intensities of urea on the bare Ag NPs and Fe_3_O_4_@C@Ag substrates are compared in Fig. 5e and f at concentrations of 10^−4^ M and 10^−6^ M. It is evident that the Fe_3_O_4_@C@Ag substrate exhibits superior sensing performance compared to the bare Ag NPs. Additionally, the EF value was calculated to further highlight the sensitivity of the Fe_3_O_4_@C@Ag substrate towards urea (detailed methods for calculating EF are presented in the ESI†). The results indicate that the Fe_3_O_4_@C@Ag SERS substrate achieves an EF of up to 3.67 × 10^6^ times, while the bare Ag NPs substrate demonstrates a modest EF of only 984 times. The combination of two key features – “dense hotspot density” and “accessible hotspots” – in Fe_3_O_4_@C@Ag likely plays a pivotal role in its superior performance compared to bare Ag NPs. The limited interaction of bare Ag NPs with urea, as demonstrated through adsorption experiments, results in urea molecules struggling to reach the Ag surface and hotspots, thus yielding lower sensor efficiency. In contrast, the carbon layer beneath the Ag layer in Fe_3_O_4_@C@Ag helps attract urea molecules to the hotspots generated by Ag on the surface. This, combined with the dense hotspot density not only on a single Fe_3_O_4_@C@Ag particle but also on a large area of the Fe_3_O_4_@C@Ag material, results in a significantly enhanced urea signal.

Table 1 compares the urea detection performance of Fe_3_O_4_@C@Ag nanostructures with that of recently published SERS substrates. The designed Fe_3_O_4_@C@Ag nanostructures exhibit superior sensing capabilities due to the synergistic combination of two key features: “dense hotspots density” and “accessible hotspots”. They achieve a LOD as low as 10^−9^ M, in stark contrast to other nanostructures, which only reach LODs between 10^−3^ and 10^−6^ M. Additionally, the EF value of up to 10^6^ times for the challenging-to-detect molecule urea highlights the remarkable SERS enhancement potential of this designed SERS substrate.

**Table 1:** Compare the sensing performance of urea detection using Fe_3_O_4_@C@Ag nanostructures substrate with that reported in recent studies

Substrate material	LOD	EF	Reliability	Ref.
Au/Cu hybrid nanostructure arrays	1.0 × 10^−3^ M	—	9.5%	25
Ag–Au compound	1.0 × 10^−3^ M	—	—	31
Ag dendrite	3.3 × 10^−3^ M			44
TiO_2_ nanofilms	4.23 × 10^−3^ M	—	4.51%	45
Au@Ag NPs	0.83 × 10^−5^ M	—	—	46
Ag/TiO_2_	4.6 × 10^−6^ M	—	8.53%	32
Fe_3_O_4_@C@Ag	5.68 × 10^−9^ M	3.67 × 10^6^	9.20%	This work

**Fig. 6:**
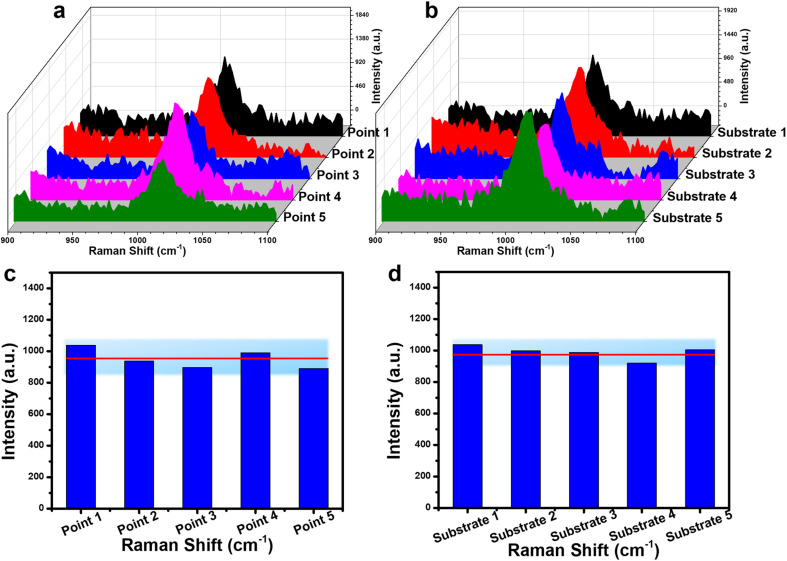
Assessment of repeatability through SERS spectra from 5 different points on the same substrate (a); evaluation of reproducibility through SERS spectra from 5 substrates across 5 different preparations (b); SERS intensity at the peak of 1010 cm^−1^ at 5 points (c) and on 5 substrates (d).

The reliability of the Fe_3_O_4_@C@Ag substrate for urea detection was evaluated through two key parameters: repeatability and reproducibility. Repeatability was assessed by collecting SERS spectra from five different points on a single SERS substrate at a urea concentration of 10^−6^ M. The results, depicted in Fig. 6a and c, demonstrate that the SERS signals from these five distinct points are remarkably consistent. Quantitatively, the RSD value was calculated to be 9.20% (detailed calculations are provided in the ESI†). Reproducibility was examined by measuring the SERS spectra on five separate substrates across five different preparations at the same concentration of 10^−6^ M (Fig. 6b and d). The results indicate that the Fe_3_O_4_@C@Ag substrate exhibits strong reproducibility, with an RSD value of 9.58%. Consequently, with both repeatability and reproducibility RSD values significantly below 10%, the Fe_3_O_4_@C@Ag nanostructures not only demonstrate ultra-high sensitivity but also provide excellent reliability in detecting urea.

### 3.3. Practicability of a Fe_3_O_4_@C@Ag nanostructure-based SERS sensor for urea detection

**Fig. 7:**
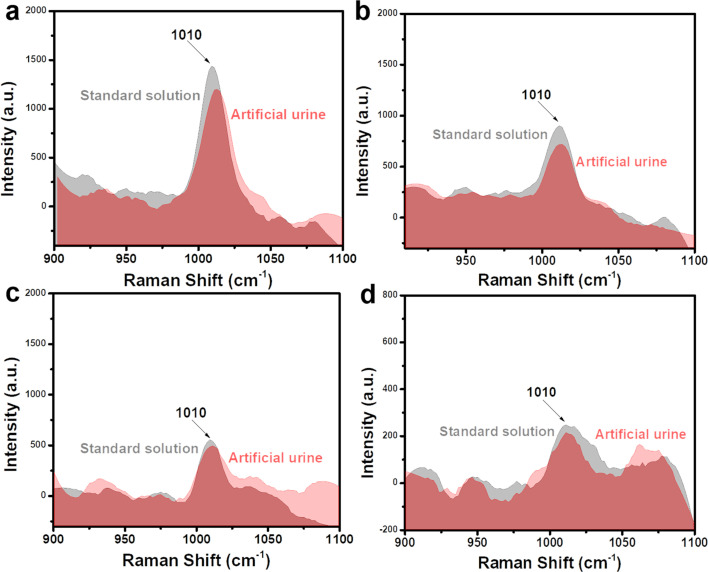
Comparison of SERS signals of urea obtained from artificial urine sample with the standard solution at concentrations of 10^−5^ M (a), 10^−6^ M (b), 10^−7^ M (c), and 10^−8^ M (d).

To investigate the practicability of a Fe_3_O_4_@C@Ag nanostructure-based SERS sensor for urea detection, we analyzed the presence of urea in two representative real sample environments corresponding to two important applications: early diagnosis and water quality analysis. These included artificial urine and tap water samples, with the preparation details outlined in Section 2.4. Four concentrations within the linear detection range (10^−5^, 10^−6^, 10^−7^, and 10^−8^ M) were selected for this experiment. Artificial urine and tap water samples containing urea at these concentrations were prepared. These real samples were used directly for SERS signal acquisition without any additional preprocessing steps. The SERS substrates were prepared in the same manner as for the standard solution experiments. For the artificial urine, droplets containing urea at different concentrations were directly deposited onto the SERS substrate and dried at room temperature. Subsequently, SERS signals were collected from these substrates, and the results are presented in Fig. 7, showing a comparison with the signal intensity obtained from standard solutions. Overall, urea signals from artificial urine samples at the prepared concentrations were still observed, with a slight reduction in intensity compared to standard solutions. At a concentration of 10^−5^ M (Fig. 7a), the characteristic peak of urea in the artificial urine sample was clearly observed, albeit with a slightly reduced intensity compared to the standard solution. This reduction could be attributed to the presence of other components in the artificial urine sample. However, these interfering factors did not significantly affect the urea signals obtained. At lower concentrations of 10^−6^, 10^−7^, and 10^−8^ M (shown in Fig. 7b–d, respectively), the characteristic urea peaks remained detectable. This demonstrates the Fe_3_O_4_@C@Ag nanostructure-based SERS sensor's excellent capability for detecting urea even in complex environments like artificial urine. The detected urea concentrations were calculated based on the intensity of the 1010 cm^−1^ peak and the linear equation *y* = 5.56 + 0.49*x* obtained from the standard solution experiments. The results are shown in Table 2, and the recovery values were calculated, showing excellent results ranging from 90% to 98%. These findings highlight the good practicality of the Fe_3_O_4_@C@Ag nanostructure-based SERS sensor for detecting urea in artificial urine. With its ultrasensitive sensing performance and demonstrated practicality for detecting urea in artificial urine, the Fe_3_O_4_@C@Ag nanostructure-based SERS sensor shows great potential for applications in early diagnosis or trace-level urea detection.

**Table 2:** Urea concentration detected by Fe_3_O_4_@C@Ag nanostructure-based SERS sensor in artificial urine and tap water samples, along with their calculated recovery values

Samples	Spiked (M)	Detected at 1010 cm^−1^ (M)	Recovery (%)
Artificial urine	10^−5^	9.20 × 10^−6^	94
	10^−6^	9.05 × 10^−7^	90
	10^−7^	9.80 × 10^−8^	98
	10^−8^	9.55 × 10^−9^	95
Tap water	10^−5^	9.40 × 10^−6^	94
	10^−6^	9.60 × 10^−7^	96
	10^−7^	9.90 × 10^−8^	99
	10^−8^	9.70 × 10^−9^	97

**Fig. 8:**
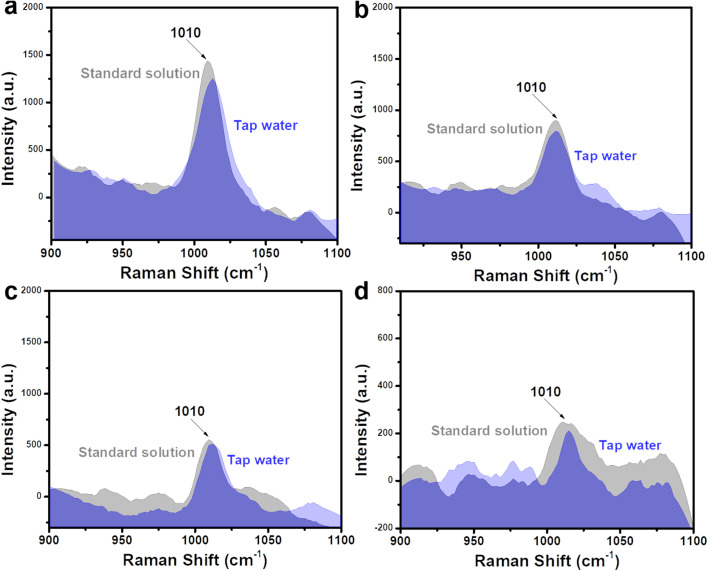
Comparison of SERS signals of urea obtained from tap water sample with the standard solution at concentrations of 10^−5^ M (a), 10^−6^ M (b), 10^−7^ M (c), and 10^−8^ M (d).

The ultrasensitive sensing performance of Fe_3_O_4_@C@Ag nanostructures was further leveraged to analyze trace-level urea residues in tap water used for daily consumption. Similar to the artificial urine sample, the SERS sensing results are presented in Fig. 8, with four concentration points: 10^−5^ M (Fig. 8a), 10^−6^ M (Fig. 8b), 10^−7^ M (Fig. 8c), and 10^−8^ M (Fig. 8d). The characteristic peak of urea is clearly observed at all four concentrations. The recovery values (Table 2) ranged from 94% to 99%, indicating the good practicality of the Fe_3_O_4_@C@Ag nanostructure-based SERS substrate. Such ultrasensitive detection performance and high practicality highlight the value of this SERS sensor for water quality monitoring, particularly regarding urea residues. The daily consumption of water containing even low levels of urea poses significant health risks, emphasizing the importance of effective monitoring.

## 4. Conclusions

This study successfully synthesized Fe_3_O_4_@C@Ag as a SERS substrate to address the challenges posed by urea, a low-Raman cross-section molecule crucial for disease diagnosis and residual analysis. The substrate integrates two key features: “dense hotspot density” and “accessible hotspots”, enabling exceptional performance in detecting urea. The achievement include (i) a limit of detection of 5.68 × 10^−9^ M, (ii) a broad linear detection range from 10^−4^ to 10^−9^ M, (iii) a high linearity coefficient of 0.98, (iv) a high enhancement factor of 3.67 × 10^6^, and (v) high reliability. Compared to conventional techniques like enzymatic assays and HPLC, which struggle with low sensitivity and matrix interference, the SERS-based approach in this study offers higher sensitivity, a broader detection range, and rapid, label-free analysis with minimal sample preparation. The Fe_3_O_4_@C@Ag substrate outperforms traditional SERS materials like Ag and Au composites by combining dense hotspots and efficient urea molecule attraction, enabling superior sensing performance. Additionally, the practicability of the Fe_3_O_4_@C@Ag substrate was demonstrated using artificial urine and tap water samples. The substrate reliably detected urea across concentrations as low as 10^−8^ M, achieving good recovery rates between 90% and 99%, even in complex sample matrices. However, the multi-step fabrication process may hinder scalability, and further studies are needed to evaluate its effectiveness for other low Raman cross-section molecules. The SERS Fe_3_O_4_@C@Ag nanostructures have further expanded the application potential of the SERS technique for low Raman cross-section molecules like urea. These findings also pave the way for new SERS-based analyses and diagnostics related to this important molecule, urea.

## Data availability

The data that support the findings of this study are available from the corresponding author upon reasonable request. All experimental data, including the characterization of the Fe_3_O_4_@C@Ag nanostructures and the detection results of urea biomolecules, are included within the manuscript and its ESI.†

## Author contributions

Q. D. Mai: conceptualization, methodology, investigation, formal analysis, data curation, supervision, writing – original draft; D. T. H. Trang: formal analysis, investigation, validation; N. T. Loan: validation, investigation; D. T. Linh: validation, formal analysis, investigation; N. T. Thanh: validation, investigation; B. H. Nhung: validation, investigation; O. V. Hoang: validation, investigation; N. X. Quang: validation, investigation; T. N. Bach: validation, investigation; A. T. Pham: methodology, supervision; A. T. Le: conceptualization, methodology, supervision, project administration, writing – review & editing.

## Conflicts of interest

The authors declare that they have no known competing financial interests or personal relationships that could have appeared to influence the work reported in this paper.

## Supplementary Material

RA-015-D4RA07487D-s001
