# Supplementary material for: Unlocking new possibility of Fe3O4@C@Ag nanostructures as an advanced SERS substrate for ultrasensitive detection of low-cross-section urea biomolecules
Source: RSC Adv. 2025 Jan 6;15(1):579–92. doi: 10.1039/d4ra07487d (PMC11702894; doi:10.1039/d4ra07487d)
Supplement: RA-015-D4RA07487D-s001 [file RA-015-D4RA07487D-s001.pdf]

Supplementary Information (SI) for RSC Advances.
This journal is © The Royal Society of Chemistry 2024

## Supporting Information

# Unlocking New Possibility of $Fe_3O_4$@C@Ag Nanostructures as an Advanced SERS Substrate for Ultrasensitive Detection of Low-Cross-Section Urea Biomolecules

Quan-Doan Mai[a,*,1], Dang Thi Hanh Trang[a,1], Dong Thi Linh[a], Nguyen Trung Thanh[b], Bui Hanh Nhung[a,b], Ong Van Hoang[c,a], Ta Ngoc Bach[d], Nguyen Quang Hoa[e], Anh-Tuan Pham[a,f], Anh-Tuan Le[a,b,**]

[a]*Phenikaa University Nano Institute (PHENA), Phenikaa University, Hanoi 12116, Vietnam*

[b]*Faculty of Materials Science and Engineering, Phenikaa University, Hanoi 12116, Vietnam*

[c]*University of Transport Technology, Trieu Khuc, Thanh Xuan District, Hanoi, Viet Nam*

[d]*Institute of Materials Science (IMS), Vietnam Academy of Science and Technology, 18 Hoang Quoc Viet, Hanoi 10000, Vietnam*

[e]*Faculty of Physics, VNU University of Science, Vietnam National University, Hanoi, Thanh Xuan, Hanoi, Vietnam*

[f]*Faculty of Biotechnology, Chemistry and Environmental Engineering, Hanoi 12116, Vietnam*

Corresponding authors:

*doan.maiquan@phenikaa-uni.edu.vn (Q.D. Mai)

**tuan.leanh@phenikaa-uni.edu.vn (A.T. Le)

[1] Q.D. Mai and D.T.H. Trang contributed equally to this work

**Calculation of limit of detection (LOD)**

The standard curve of linear detecting range was given as:

$$Y = A + B \times Log(X) \tag{1}$$

where A and B are intercept and slope of regression equation obtained through the plot of the logarithmic SERS intensity (Y) – logarithmic concentration (X).

The LOD is calculated using the following equation(1):

$$LOD = 10^{\left[\left(Y_{blank} + 3SD\right)/Y_{blank} - A\right]/B} \tag{2}$$

where $Y_{blank}$ and SD are the SERS signal and the standard deviation of blank sample, respectively.

SD is calculated via the well-known formula:

$$SD = \sqrt{\frac{1}{n-1} \times \sum_{i}^{n} (x_i - x_{average})^2} \tag{3}$$

where $x_i$ if the "i" sample of the series of measurements, $x_{average}$ is the average value of SERS signal obtained from the blank sample repeated n times.

**Calculation of enhancement factor (EF)**

The EF value is calculated according to the well-established equation, which was employed in several published studies(2, 3):

$$EF = \frac{I_{SERS}}{I_{Raman}} \times \frac{N_{bulk}}{N_{surface}} \tag{4}$$

where $I_{Raman}$ is the Raman signal intensity of the 1020 $cm^{-1}$ peak of the urea collected directly from the powder sample (the result is presented in Figure S5); $I_{SERS}$ is the SERS intensity of the 1010 $cm^{-1}$ peak of urea on the SERS substrate based on Ag NPs and $Fe_3O_4$@C@Ag; and $N_{bulk}$ is the number of analyte molecules that are probed on the Raman spectrum of urea powder, while $N_{surface}$ is the number of analyte molecules probed using SERS.

$N_{bulk}$ can be calculated following:

$$N_{bulk} = \frac{A_{laser} \times h \times \rho}{M} \times N_A$$

(5)

where $A_{laser}$, h, ρ and m are the laser spot area, the focal length, the density of the solid analyte and its molecular weight, respectively; and $N_A$ is the Avogadro number.

$N_{surface}$ can be expressed as:

$$N_{surface} = \frac{C \times V}{A_{substrate}} \times N_A \times A_{laser} \qquad (6)$$

where C, V, $A_{substrate}$ are the concentration, the volume drop-casted of the analyte, and the area of the substrate, respectively; $N_A$ is the Avogadro number; and $A_{laser}$ is the laser spot area.

Thus EF can be calculated as:

$$EF = \frac{I_{SERS}}{I_{Raman}} \times \frac{N_{bulk}}{N_{surface}} = \frac{I_{SERS}}{I_{Raman}} \times \frac{h \times \rho \times A_{substrate}}{M \times C \times V}$$

(7)

In our case, $I_{Raman}$ value is determined by the intensity of the peak at 1020 $cm^{-1}$ in the Raman spectrum of urea in its powdered form, $I_{SERS}$ value is obtained from the intensity of the peak at

1010 $cm^{-1}$ in the SERS spectrum of urea at a concentration of $10^{-8}$ M for $Fe_3O_4$@C@Ag substrate and $10^{-4}$ M for Ag NP substrate, h = 2 μm, $\rho_{urea}$ = 1.32 g/$cm^3$; $M_{urea}$ = 60 g/mol; $A_{substrate}$ = 4 $mm^2$, V = 5 μL.

**Calculation of relative standard deviation (RSD)**

The RSD value of repeatability and reproducibility is calculated via the well-known formula:

$$RSD = \frac{SD \times 100}{x_{average}} \quad (8)$$

where SD is the standard deviation that calculates using equation 3 and $x_{average}$ is the average value of SERS signal obtained from each measurement.

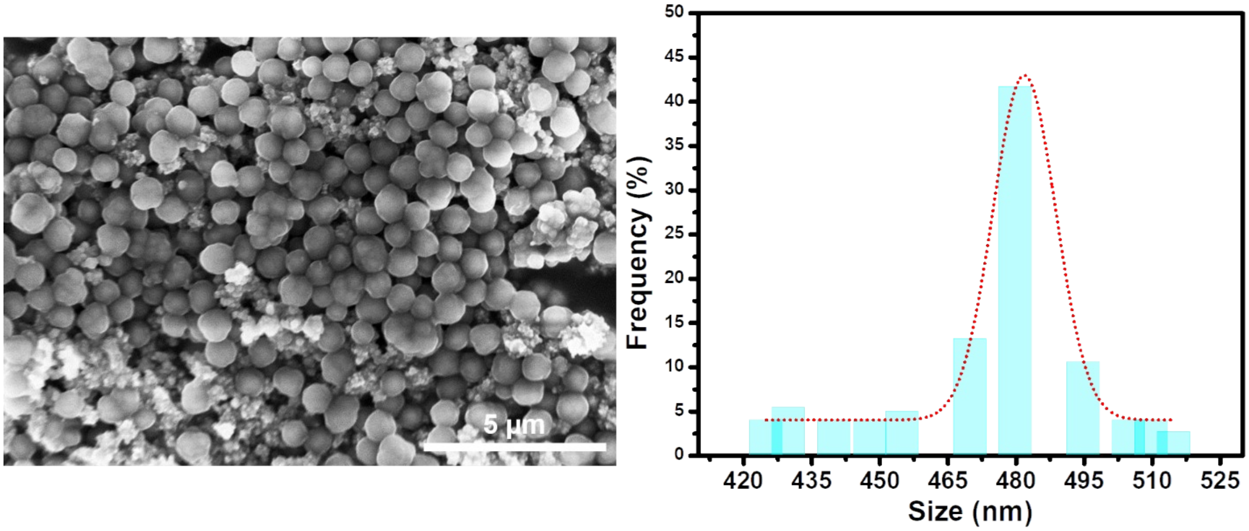


**Figure S1. FE-SEM images and the size distribution of $Fe_3O_4$@C materials.**

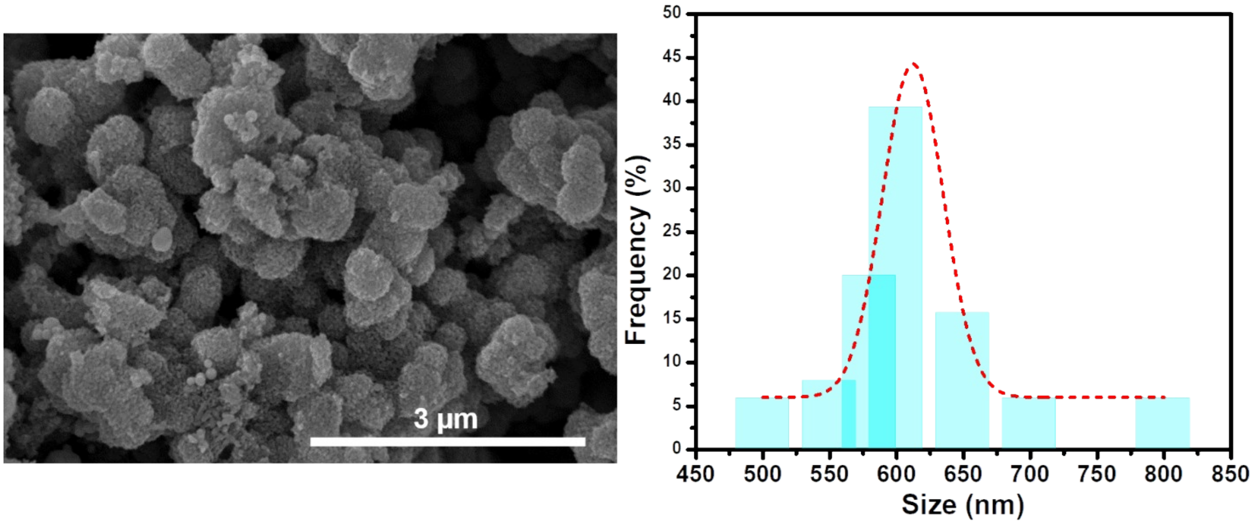


**Figure S2. FE-SEM images and the size distribution of $Fe_3O_4$@C@Ag materials.**

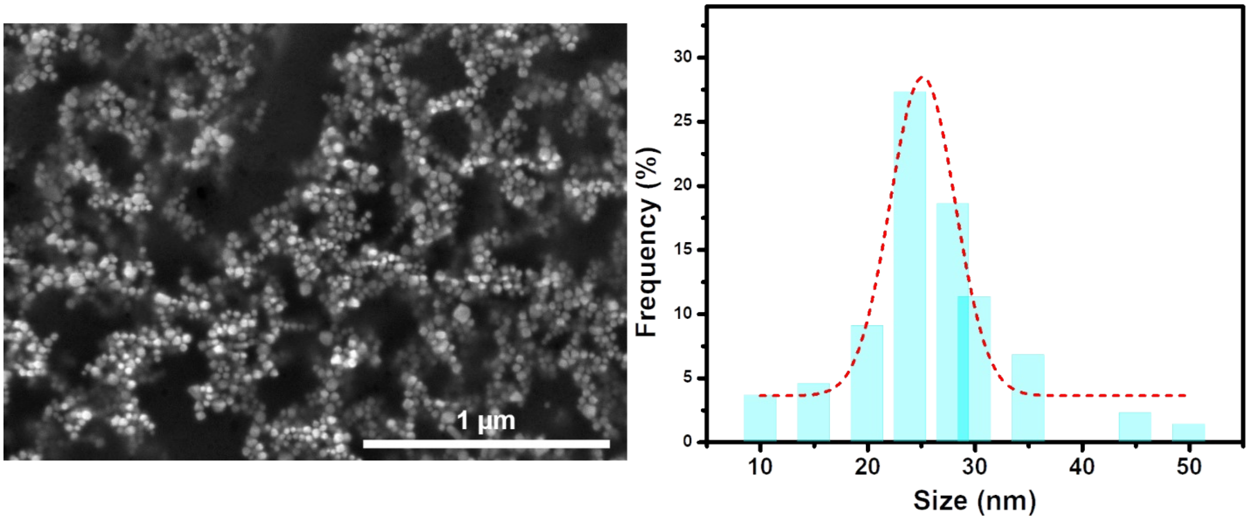


**Figure S3. FE-SEM images and the size distribution of Ag nanoparticles.**

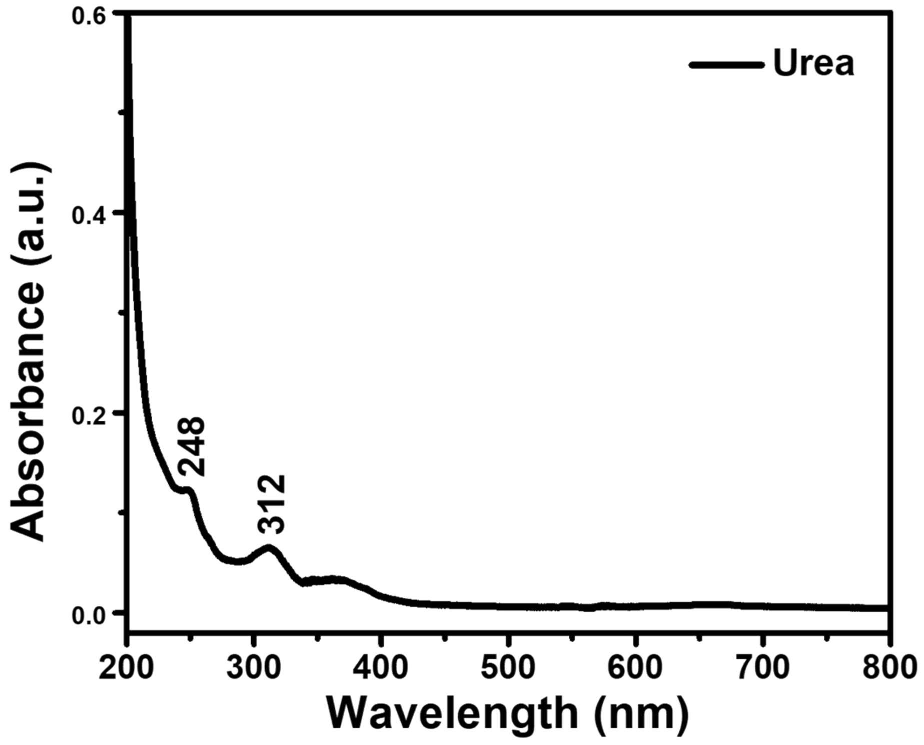


**Figure S4. UV-Vis spectrum of urea at a concentration of $10^{-4}$ M.**

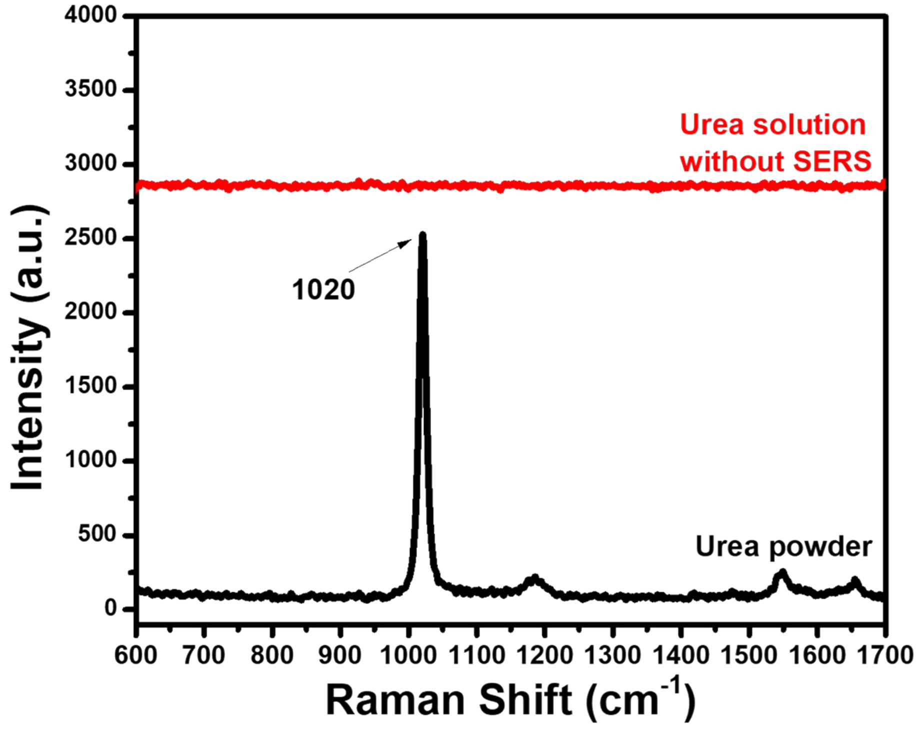

**Figure S5. Raman spectra of urea powder and urea solution at a concentration of $10^{-3}$ M on the aluminum substrate in the absence of SERS material.**

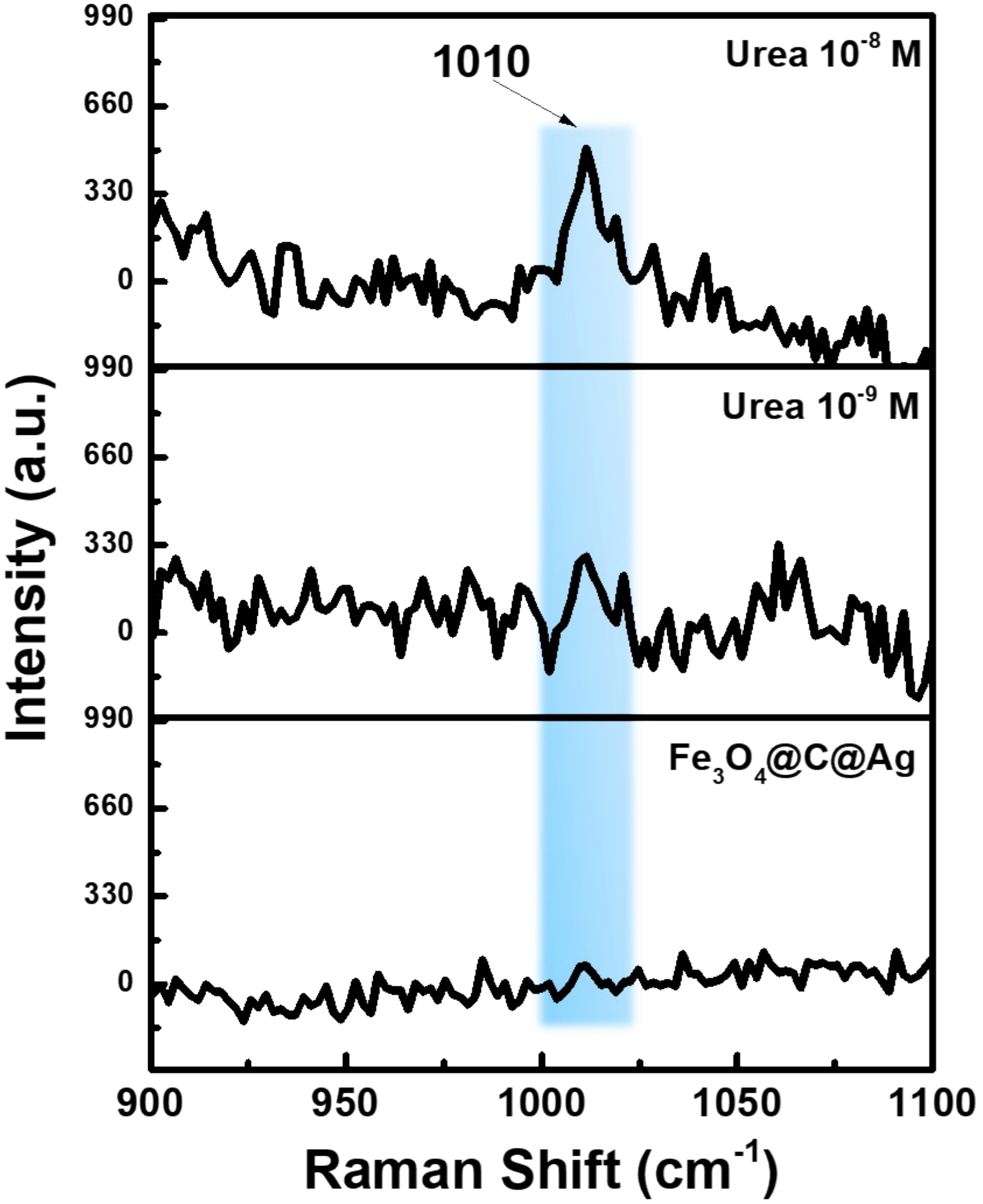


**Figure S6. The Raman spectrum of the bare $Fe_3O_4$@C@Ag substrate and the SERS spectra of urea at concentrations of $10^{-8}$ M and $10^{-9}$ M.**

**References**

1. Chen R, Shi H, Meng X, Su Y, Wang H, He Y. Dual-amplification strategy-based SERS chip for sensitive and reproducible detection of DNA methyltransferase activity in human serum. Analytical chemistry. 2019;91(5):3597-3603.

2. Le Ru EC, Blackie E, Meyer M, Etchegoin PG. Surface enhanced Raman scattering enhancement factors: a comprehensive study. The Journal of Physical Chemistry C. 2007;111(37):13794-13803.

3. Fu WL, Zhen SJ, Huang CZ. One-pot green synthesis of graphene oxide/gold nanocomposites as SERS substrates for malachite green detection. Analyst. 2013;138(10):3075-3081.
